# Regulation of Extracellular Vesicle-Mediated Immune Responses against Antigen-Specific Presentation

**DOI:** 10.3390/vaccines10101691

**Published:** 2022-10-10

**Authors:** Yasunari Matsuzaka, Ryu Yashiro

**Affiliations:** 1 Division of Molecular and Medical Genetics, Center for Gene and Cell Therapy, The Institute of Medical Science, The University of Tokyo, Minato-ku, Tokyo 108-8639, Japan; 2 Administrative Section of Radiation Protection, National Institute of Neuroscience, National Center of Neurology and Psychiatry, Kodaira 187-8551, Tokyo, Japan; 3 Department of Infectious Diseases, Kyorin University School of Medicine, 6-20-2 Shinkawa, Mitaka-shi 181-8611, Tokyo, Japan

**Keywords:** dendritic cells, extracellular vesicles, immune response, major histocompatibility complex, mesenchymal stem cell, microRNAs, tumour vaccination

## Abstract

Extracellular vesicles (EVs) produced by various immune cells, including B and T cells, macrophages, dendritic cells (DCs), natural killer (NK) cells, and mast cells, mediate intercellular communication and have attracted much attention owing to the novel delivery system of molecules in vivo. DCs are among the most active exosome-secreting cells of the immune system. EVs produced by cancer cells contain cancer antigens; therefore, the development of vaccine therapy that does not require the identification of cancer antigens using cancer-cell-derived EVs may have significant clinical implications. In this review, we summarise the molecular mechanisms underlying EV-based immune responses and their therapeutic effects on tumour vaccination.

## 1. Introduction

**Figure 1:**
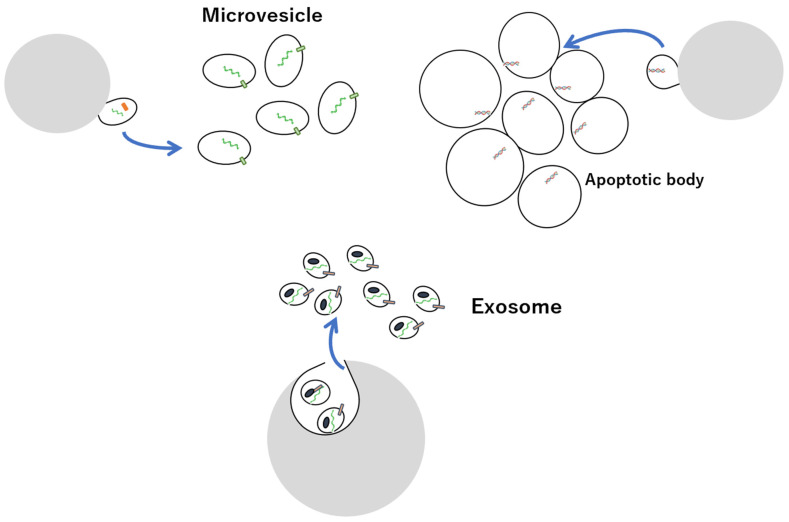
Overview of EVs exosomes, microvesicles, and apoptotic bodies.

Extracellular vesicles (EVs) are lipid bilayer structures secreted by living cells and are classified into either exosomes, microvesicles (MVs), or apoptotic bodies, based on the intracellular production mechanism and size (Figure 1) [1,2,3,4,5,6,7,8,9,10,11,12,13,14,15,16,17,18,19,20,21,22,23,24,25,26,27,28,29,30,31,32,33,34,35,36,37,38,39,40,41,42,43,44,45,46,47,48,49,50,51,52,53,54,55,56]. Apoptotic bodies are released from apoptotic cells, whereas exosomes and MVs are released from healthy cells. Exosomes are endosomal membrane-derived vesicles, approximately 50–150 nm in size, formed during endocytosis and secreted by almost all types of cells, and are present in large numbers in body fluids such as blood, urine, cerebrospinal fluid, tears, and saliva. Their main constituents are lipids, proteins, and nucleic acids, including microRNAs (miRNAs), messenger RNA (mRNA), and DNA derived from secretory cells transferred to other cells [12,57,58,59,60,61,62,63,64,65,66,67,68,69,70,71,72,73,74,75,76,77,78,79,80,81,82,83,84,85,86,87,88,89,90,91,92,93,94,95,96,97,98,99,100,101,102,103,104,105,106,107,108,109,110,111,112,113,114,115,116,117,118,119,120,121,122,123,124,125,126,127,128,129,130,131,132,133,134,135,136,137,138,139]. Exosomes are involved in various physiological activities, such as immune regulation, neurodegeneration, and cancer development, as well as in the onset of disease mediated by intercellular communication involving the uptake of EVs into recipient cells [57,106,119,140,141,142,143,144,145,146,147,148,149,150,151,152,153,154,155,156,157,158,159,160,161,162,163,164,165,166,167,168,169,170,171,172,173,174,175,176,177,178,179,180,181,182,183,184,185,186,187,188,189,190,191,192,193,194]. Hence, preventive, diagnostic, and therapeutic strategies that target or use exosomes are likely to be effective and have significant potential in a clinical setting. Exosomes contain multivesicular body-related proteins, such as apoptosis-linked gene 2-interacting protein X (Alix); tumour susceptibility gene 101 (TSG101); the endosomal sorting complex required for transport complex (in late endosomes); heat shock proteins, such as HSP70 and HSP90; proteins involved in intracellular transport, such as Rab GTPase; and transmembrane protein family tetraspanins, such as CD9, CD61, and CD81, in addition to endosome membrane-derived lipids, such as cholesterol and sphingomyelin, whose expression levels differ based on the cell type from which they are secreted [195,196,197,198,199]. Exosomes are classified based on size; ~35 nm particles are referred to as exomeres, 60–80 nm particles as small exosomes, and 90–120 nm particles as large exosomes [200,201,202,203,204,205,206,207], all of which exhibit different expression patterns for proteins, lipids, nucleic acids, and N-glycans. 

Conversely, MVs are vesicles with a wide range of sizes (100–1000 nm) and bud directly from the cell membrane into the outside of the cell [208,209]. Although MVs production mechanisms differ, many MVs are similar to exosomes, rendering them indistinguishable. Apoptotic bodies, micrometres in size, are particles in which apoptotic cells are displayed from the membrane and can be separated via low-speed centrifugation, hence are distinguishable from MVs and exosomes [210,211,212,213].

EVs are produced by various immune cells, including B and T cells, macrophages, dendritic cells (DC), natural killer (NK) cells, mast cells, and thymocytes [164,214,215,216,217,218] and are rich in proteins with immune functions, such as antigen-presenting molecules (major histocompatibility complex (MHC) class I, MHC class II, CD1), adhesion molecules (CD11b, intercellular adhesion molecule 1 (ICAM-1)), and co-stimulatory proteins (CD86) [219,220,221,222]. Additionally, exosomes are involved in releasing intracellular components to the outside of cells as waste products, and through their loaded immune-related molecules, they have various immune functions, such as antigen presentation, including the priming of early T cells, differentiation of mature T cells, development of effector functions, and regulation of immune-related cells [223,224,225,226,227].

Vaccine therapy is based on antigen presentation attributed to heightened immune function of EVs produced by DCs and antigen-presenting cells (APCs) [73,228,229,230,231]. Meanwhile, EVs produced by cancer cells contain cancer antigens; hence, the development of vaccine therapy that requires no identification of cancer antigens using cancer-cell-derived EVs is possible. EVs produced from mesenchymal stem cells (MSCs) have anti-inflammatory and tissue-regeneration properties, which may facilitate the development of immunotherapy [232,233,234,235]. Moreover, the development of novel immunotherapies that utilise EV characteristics, such as easy uptake by phagocytic cells or release from cells near target tissues, is underway [236,237]. In this review, we summarise EV-based vaccine therapies and novel immunotherapies.

## 2. EV-Based Vaccine Therapy for Cancer

### 2.1. Immune Response via APCs with Functions of Chemokine

Cancer antigens and neoantigens can induce strong immune responses. When a cancer vaccine is administered, it is first taken up by APCs, including DCs that migrate to the lymph node, thereby activating antigen-specific T cells leading to the initiation of an immune response [238,239,240]. After undergoing positive and negative regulation, tumour-specific T cells exit the lymph node and migrate to the tumour site to eliminate cancer cells, although this process is strongly influenced by the immune environment created by the tumour [241,242,243]. In the cancer immune cycle, cancer antigens are released from cancer cells that have experienced cell death and are taken up by DCs [244,245]. These DCs mature, migrate to the lymph nodes, and present the captured cancer antigen to the MHCI molecules, thereby activating T cells [246,247,248,249], which in turn migrate and infiltrate the tumour tissue to recognise and eliminate tumour cells. The interaction between T cell-expressed integrin alpha L (LFA1) and ICAM-1 on the vascular endothelium initiates T cell infiltration into tumours that are suppressed by vascular endothelial growth factor A (VEGF-A), which is produced by cancer cells, further releasing new cancer antigens and continuing the cancer immune cycle [250,251,252]. In this process, the loss of beta-2 microglobulin, which is involved in the MHCI antigen presentation pathway in cancer cells, allows cancer cells to evade recognition by T cells [253,254,255]. Cancer cells in which β-catenin signalling is activated have low chemokine (C-C motif) ligand 4 production and suppress the accumulation of conventional DC (cDC1), which produces chemokine (C-X-C motif) ligand 10 (CXCL10) in the tumour, thereby hindering T cell infiltration [256,257,258]. The deletion of the phosphatase and tensin homologue (Pten) in cancer cells suppresses T cell infiltration into the tumour site [259,260,261]. In this process, interferon-γ (IFN-γ), produced by T and NK cells, suppresses tumour cell proliferation and enhances MHCI antigen presentation. Additionally, the M1 type of tumour-associated macrophages (TAMs) promotes anti-tumour immunity [262,263,264]. When cancer cells undergo immunogenic cell death, such as necrosis, high mobility group box 1, released at the same time as the antigen, acts on Toll-like receptors TLR2 and TLR4 on DCs to induce their maturation, leading to the efficient induction of anti-tumour immunity [265,266,267]. Among the DCs subsets within tumours, having a large amount of cDC1 is beneficial, since they take up cancer antigens from tumours and migrate to lymph nodes, in which chemokine expressions, such as CXCL9, 10, and CCL5, in the tumour and intratumoural environment induce stronger T cell migration and cross-prime CD8^+^ T cells [268,269,270]. Furthermore, NK cells in tumour tissues produce chemokines, such as CXCL1, CCL5, and FMS-like tyrosine kinase 3 ligands, which affect cDC1 [271]. The depletion of NK cells strongly suppresses the potentiation of immune checkpoint blockage action by interleukin (IL)-18, which regulates innate immunity, enhances the anti-tumour effect of immune checkpoint blockage through the induction of characteristic NK cells, which accumulate in the peritoneal cavity during early treatment prior to CD8^+^ T cells, and expresses CXCL1, whose depletion decreases the recruitment of CD103^+^CXCR1^+^ cDCs to the peritoneum [272,273,274].

During antigen presentation by DCs, the expression of chemokine (C-C motif) receptor 7 (CCR7) on DCs induces migration to lymph nodes, where the chemokines CXCL13, CCL19, and CCL21 play a central role in adaptive immunity by exerting their effects via the actions of CXCL13 or CCL19 and CCL21 on receptors such as CXCR5 and CCR7, respectively [275,276,277,278]. These chemokines have two important functions in lymphocytes. First, interactions between CCL21, produced in the epithelial cells of lymph nodes, and CCR7, expressed on the surface of lymphocytes, activate integrins and induce adhesive responses. As a result, lymphocytes change shape and enter the lymph nodes through intercellular spaces that constitute high endothelial venules. Second, in the parenchyma of the lymph node, stromal cells secrete CCL19 and CCL21 around T cells, thereby promoting the accumulation of T cells that strongly express CCR7 [279,280]. In contrast, CXCL13 expressed in the B cell region attracts B cells expressing CXCR5. These T and B cells in turn migrate along networks shaped by stromal cells, forming a 3D microenvironment within a lymph node, such as follicular DCs in B cell follicles and reticular fibroblasts in T cell areas.

In addition, the CD40/CD40L immune checkpoint pathway, which rescues tumour cells from apoptosis, prolongs survival and enhances the proliferation, activation, or maturation of APCs, including primarily macrophages and DCs, to produce IL-12 or IL-18 to stimulate NK cells. These, in turn, produce IFN-γ and express co-stimulatory molecules, such as CD80/CD86, ICAM-1, and CD44, which are required for the non-energetic full activation of T cells following T cell receptor stimulation [281,282].

Furthermore, regulatory T cells in lymph nodes interact with cytotoxic T-lymphocyte-associated protein 4 (CTLA-4), CD80, and CD86 on DCs to suppress T cell priming [283,284]. Anti-tumour immune responses are suppressed when cancer cells undergo tolerance-inducing cell death, such as apoptosis, during the release of cancer antigens [285]. Furthermore, cancer-associated fibroblasts (CAFs) create an immunosuppressive environment by remodelling the extracellular matrix of the tumour microenvironment [286,287,288]. In many cancer patients, one or more of these steps in the cycle are impaired, thereby preventing the induction of effective cancer immune responses.

### 2.2. EV Application of Vaccine Therapy

Therapy using EVs derived from cancer cells has gained popularity since it contains cancer antigens and induces a cancer antigen-specific immune response, which provides an anti-tumour effect [289]. Although inducing anti-tumour immunity using cancer-cell-derived EVs has been reported, the effect remains to be verified. Insufficient dynamic control of EVs and the low delivery efficiency to APCs, along with insufficient antigen presentation efficiency due to the effect of immunostimulatory agents, adjuvants, or low delivery efficiency to APCs remain major challenges. Pharmacokinetics analysis has shown that intravenously administered EVs derived from cancer cells accumulated in some tissues, such as the liver, spleen, and lungs. In the liver and spleen, it is taken up by macrophages and by vascular endothelial cells in the lungs [290,291]. Consequently, they are quick to disappear from the blood. Conversely, topical administration, which is frequently used in vaccine administration, showed that EVs are rapidly taken up by macrophages and other cells. Therefore, for the development of vaccine therapy using cancer-cell-derived EVs, it is necessary to control their kinetics, that is, continuous antigen delivery to DCs by imparting retention of EV at the administration site, thereby conferring tropism to DCs and controlling the intracellular dynamics of engulfed DC [7,292,293,294,295]. APCs, including DCs, are easy to use in large particles compared with other cell types.

Additionally, the disappearance of locally administered particles from the administration site is delayed as the size increases. This increase in size caused by EV formation aggregates may be used to confer APC tropism and retention at the administration site. The uptake of EV aggregates by DCs, in which EVs are linked by complementary strands of DNA, is increased by 2-fold, while the uptake into other cell types is reduced by approximately half. Furthermore, retention at the administration site following subcutaneous administration in mice has been shown to significantly increase due to aggregate formation. In mouse solid tumour models, EV aggregates are shown to induce anti-tumour immunity more efficiently than EVs and markedly delay tumour growth [296,297,298]. Therefore, EV aggregation is a useful kinetic control method to obtain the kinetics required for vaccine therapy.

## 3. Antigen Presentation through EVs Derived from Cancer Cells

**Figure 2:**
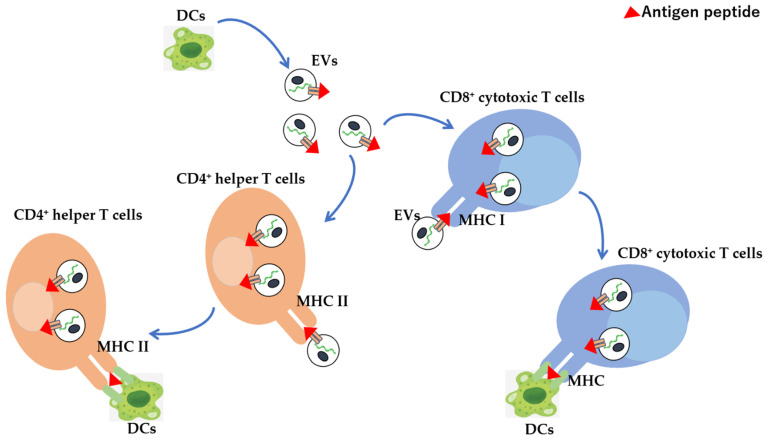
EV-mediated antigen presentation to CD4+ helper or CD8+ cytotoxic T cells from dendritic cells. DC-derived EVs, which can migrate to tumours or the spleen directly or indirectly present antigens to CD4+ helper and CD8+ cytotoxic T cells via MHC molecules, thereby inducing immune responses. Red triangle: antigen peptide.

DCs are among the most active exosome-secreting cells in the immune system, and they take up foreign substances such as bacteria that invade the body into phagosomes, fragment them into antigenic peptides, and activate T cells by placing them on MHC class II molecules, where are they presented on the cell surface [299,300]. In contrast, intracellular antigenic peptides are presented by MHC class I molecules; however, exosomes secreted by DCs contain both MHC classes I and II. By binding to antigen-specific T cell receptors of CD8^+^ cytotoxic T cells and CD4^+^ helper T cells, T cells can be activated away from DCs (Figure 2). However, since the expression of co-stimulatory molecules, which are important for T cell activation, is lower on the exosome surface than DCs surface, the activation is as low as 5–10% compared with cases where DCs are activated by direct contact with T cells. Furthermore, by carrying antigen peptide/MHC complexes, exosomes not only play a direct antigen-presenting function but deliver exosomes into other APCs. It is also possible to indirectly promote antigen presentation by passing antigens to MHC molecules within cells [301,302,303]. When antigens are presented by DCs that have endocytosed EVs derived from cancer cells, they are presented to MHC class II by degradation of the antigenic protein by endosomes, and to the MHC class I by degradation of the antigenic protein in the cytoplasm. In contrast, exosomes derived from phagocytic cells, such as macrophages, contain antigens derived from phagocytosed bacteria; it is possible to promote the efficient activation of T cells by transferring the antigen to DCs with stronger antigen-presenting ability via exosomes. When considering cancer therapy, the induction of cell-mediated immunity is desirable, where antigen presentation to MHC class I is important for this purpose. The escape of EVs from endosomes is useful for delivering endocytosed proteins to the cytoplasm, hence modifying cancer-cell-derived EVs with GALA peptide, a 30-amino-acid synthetic peptide with a glutamic acid–alanine–leucine–alanine (EALA) repeat, exerts a membrane-disrupting ability in a low-pH environment. Morishita et al. effectively illustrated the possibility to destroy lipid membranes at low pH, escape endosomes in engulfed DCs, and efficiently present cancer-cell-derived antigens to MHC class I on DCs [304,305,306]. Thus, GALA modification promotes the transport of exosome inclusions into the cytoplasm and enhances the MHC class I presentation of cancer antigens based on the control of intracellular dynamics. Therefore, since cancer-cell-derived exosomes contain cancer antigens and can induce anti-tumour immune responses, they can be used as novel cancer vaccine formulations without the identification of cancer antigens. Furthermore, DC directivity can be conferred by a DC-directing ligand. To transfer CD40L to the surface of cancer-cell-derived EVs, a CD40L-LA (Lactadherin) fusion protein in which LA has an affinity for phosphatidylserine, a lipid present in the EV membrane, was used. Liu et al. revealed that CD40L-modified EVs show an increased ability to activate DCs than unmodified EVs [307].

## 4. Antigen-Specific Immune Response in Cancer

The immune response that eliminates foreign substances such as bacteria and viruses does not attack host cells. This mechanism, referred to as “autoimmune tolerance”, is induced by eliminating harmful cells such as cancer cells and unnecessary cells in the body by phagocytes, such as macrophages and DCs [308,309]. This process prevents dead cells from releasing harmful substances and helps the surrounding tissues to maintain normal function.

However, if the elimination of dead cells is delayed, or the types of cells that eat dead cells change, immune tolerance breaks down. Cancer vaccines for cancer immunotherapy are composed of cancer antigens and adjuvants, which induce antigen-specific cytotoxic T cells through APC presentation and promote the exclusion of cancer cells.

When DCs that differentiate in vitro are loaded with tumour antigens and reinjected into the patient, they present antigens to naïve T cells. CD8^+^ naïve T cells are activated to differentiate into cytotoxic T cells, which results in antigen-specific antitumour activity. Conversely, CD4^+^ naïve T cells differentiate into helper T cells to assist cytotoxic T cells, which present antigens circulating in the bloodstream and improve disease conditions and the suppression of disease progression, even in patients with distant metastases. Further, some anti-tumour effects of DCs are retained in memory T cells. The activation of DCs that have taken up cancer antigens is important for the induction of cancer antigen-specific immune responses. Therefore, developing a method to deliver cancer-cell-derived EVs and adjuvants to the same DCs will have significant therapeutic implications. The modification of cancer-cell-derived exosomes containing cancer antigens with an adjuvant induces potent anti-tumour immunity through the simultaneous delivery of cancer antigens and adjuvants to DCs. Morishita et al. reported that cancer-cell-derived EVs were modified with an SAV-LA fusion protein between biotin-binding protein streptavidin (SAV) and LA, reacting with a biotin derivative of CpG DNA, an immunostimulatory nucleic acid [310]. This nucleic acid has attracted attention as an adjuvant consisting of unmethylated cytosine and guanine (CpG), frequently present in the genomic DNA (CpG-DNA) of bacteria and viruses for host defence in mammalian innate immunity, in which two sequences, K type and D type, are particularly effective for CpG-DNA. The K type mainly induces the proliferation of B cells and production of cytokines such as IL-6, while the D type induces the production of the type I interferon from plasmacytoid DCs. The mechanism underlying immune activation involves the binding of CpG-DNA to TLR9 in the endosome, which induces the nuclear factor kappa B (NF-κB) pathway through the adaptor molecule myeloid differentiation primary response gene 88, thereby inducing the production of various cytokines [311,312,313].

When CpG DNA-modified EV was added, uptake into DCs increased compared to the addition of CpG DNA alone. Therefore, CpG DNA modification of EVs enables the simultaneous delivery of cancer antigens and adjuvant CpG DNA to the same cell [308]. Additionally, DCs supplemented with CpG DNA-modified EVs showed a higher cytokine production than those with CpG DNA or EVs alone. Furthermore, in an in vivo experimental system using tumour-bearing mouse models, CpG DNA-modified EVs can induce cancer antigen-specific cellular and humoral immune responses and significantly suppress tumour growth and metastasis to the lungs, thereby prolonging survival [314,315,316]. Therefore, the CpG DNA modification of cancer-cell-derived EVs enables the simultaneous delivery of cancer antigen and adjuvants to the same DCs and the induction of anti-tumour immunity.

## 5. DC-Derived EVs as Prospective Vaccines

DCs, which serve as a link between innate and adaptive immunity, are involved in the initiation and suppression of immune responses. Antigen presentation to naïve cytotoxic and helper T cells is performed through MHC classes I and II molecules, respectively. Molecules contained in DC-derived EVs are MHC class I/II and CD86 proteins, which are capable of mediating antigen presentation to CD8^+^ and CD4^+^ T cells and the subsequent proliferation of T cells [317].

CD1a, b, c, or d proteins are involved in the presentation of lipid antigens, while ICAM-1 plays an important role in regulating DC-T cell communication, where ICAM-1 can either promote the uptake of DC-derived EVs by target DCs or the interaction of T cells with DCs that retain DC-derived EVs on their outer surface as ligands for Mac1 integrin (CD11b/CD18) and lymphocyte function-associated antigen 1 (LFA1, CD11a/CD18). Additionally, DC-derived EVs are rich in tetraspanins, including CD9, CD37, CD53, CD63, CD81, and CD82, which regulate DC interactions. Thus, DC-derived EVs can induce cellular and humoral immunity by antigen presentation via DCs that have incorporated them. A clinical trial for vaccine therapy using DC-derived EVs has been conducted, showing that although its safety has been confirmed, its therapeutic effect is limited. Ovalbumin (OVA) has been previously used as a model antigen; EVs were recovered from DCs spiked with OVA, along with LPS and IFN-γ as activators of DCs, where it carried OVA, in addition to MHC class I, antigen presentation, and co-stimulatory molecules such as CD86 [318,319,320]. As DC-derived EVs have a strong ability to activate immune cells, the addition of macrophages and DCs further exacerbates the activation of these cells. These DC-derived EV-loaded DCs can present antigens to T cells, while the DC-derived EVs can directly present antigens to T cells. Furthermore, the administration of DC-derived EVs to tumour-bearing model mice, established by transplanting OVA-expressing cancer cells, induced cellular and humoral immunity specific to the loaded OVA antigen thereby displaying anti-tumour effects. Thus, EVs recovered from activated DCs have properties that contribute to vaccine therapy, while the optimisation of the activation state of DCs may produce EVs with enhanced activity.

## 6. Application of MSC-Derived EVs in Modulating the Immune Response

MSCs are fibroblast-like progenitor cells recovered from the bone marrow, adipose tissue, and umbilical cord. They have adipogenic, chondrogenic, and osteogenic differentiation potential [321,322,323,324,325,326,327,328]. MSCs do not express human leukocyte antigen (HLA) class II antigens; therefore, they are not only less immunogenic in allogeneic transplantation but are also capable of suppressing the function of various immune cells. To date, clinical trials have been conducted using MSCs as novel therapeutic agents to treat various diseases, including acute diseases, such as ischaemic stroke and myocardial infarction. MSCs are also intended anti-inflammatory therapy for diseases caused by uncontrolled inflammatory reactions, such as acute graft-versus-host disease (GvHD) and Crohn’s disease [329,330,331,332].

Initially, MSCs were characterised by their cell differentiation potential and their direct interaction with immune cells. However, it has become clear that the immunoregulatory ability of MSCs is based on paracrine action; EVs containing marker proteins, such as CD9, CD81, and ALIX, at approximately 100 nm from MSC culture supernatant have therapeutic effects on disease model mice. Furthermore, in a clinical trial where EVs recovered from steroid-refractory GvHD patients, increasing the dose of MSC-derived EVs resulted in a long-term reduction in GvHD symptoms and reduced steroid dose. The addition of MSC-derived EVs to patient-derived peripheral blood cells suppressed the secretion of the inflammatory cytokines IL-1β, tumour necrosis factor-alpha (TNFα), and IFNγ [333]. Moreover, MSC-derived EVs comprised anti-inflammatory cytokines, transforming growth factor-β (TGF-β), IL-10, and HLA-G, further improving GvHD symptoms and chronic kidney disease. Notably, the levels of TGF-β and IL-10 in peripheral blood were significantly elevated relatively early following administration of MSC-derived EVs, and their levels remained high even after one year. Conversely, the inflammatory cytokine TNFα was significantly suppressed by the administration of MSC-derived EVs.

In addition to GvHD, MSC therapy may be clinically applied to other diseases such as severe heart failure and type I diabetes [334,335,336,337]. The administration of MSCs improves heart failure in the disease model mice, and adiponectin produced by adipocytes increases the exosomes produced by MSCs, thereby promoting their therapeutic effects. Furthermore, in a mouse model that developed diabetes by inhibiting the binding of Programmed Cell Death Protein 1/Programmed Cell Death Ligand 1 (PD-L1), immune cell infiltration into the space between pancreatic insulin-producing cells was observed using immune checkpoint inhibitors. In particular, there was a marked increase in cytotoxic macrophages that destroyed pancreatic insulin-producing cells. The administration of human adipose tissue-derived MSCs to these mice suppressed the infiltration of immune cells and the onset of diabetes. Furthermore, following the administration of MSCs, a marked increase in MSC-derived exosomes in the blood of mice was observed, suggesting that humoral factors such as exosomes may be involved in the suppression of diabetes development. The infiltration of immune cells, such as cytotoxic macrophages, was also observed in human islets following the administration of immune checkpoint inhibitors. Thus, humoral factors, such as exosomes produced by human adipose tissue-derived MSCs, suppress the onset of type 1 diabetes induced by immune checkpoint inhibition.

## 7. EV-Based Immunotherapy

### 7.1. Immune-Related Molecules Loaded into Exosomes

EVs not only contain protein antigens but also secretory cell-derived mRNAs and non-coding RNAs, especially miRNAs, and their functions have attracted much attention. These RNAs are protected by the lipid bilayer membrane of exosomes, and thus are not degraded by RNases and remain stable in the blood and body fluids. While there are RNAs detected in both exosomes and their secreting cells, there are others that can only be detected in either, suggesting the existence of a mechanism to selectively incorporate specific RNAs into exosomes taken up by target cells fused with the endosomal membrane. This releases trapped RNA into the target cell cytoplasm. In this study, the released mRNAs are translated into proteins, miRNAs suppress the translation of target genes, while exosomes regulate gene expression in target cells. Immune cells that encounter a foreign object horizontally transmit their activation state to cells that have not yet encountered it through RNA. In addition, various immune-related molecules are loaded into exosomes, which in turn regulate the immune responses. For example, exosomes derived from cytotoxic T cells and NK cells carry TNF family proteins, such as Fas ligand, TRAIL, and CD40 ligand, and induce apoptosis in target cells [338]. Similarly, some cancer cells release exosomes loaded with the Fas ligand and TRAIL, which induce apoptosis in immune cells, thereby escaping immune attack. The TNF family proteins are produced in a membrane-bound form and cleaved by a membrane-type metalloprotease to become soluble. Apoptosis-inducing activity is mainly mediated by the membrane form, while the activity of the soluble form is weak. Moreover, TNF family proteins on exosomes are stable without being cleaved by membrane metalloproteases and have strong apoptosis-inducing activity by forming trimers through the membrane. The exosome-mediated transport of TNF family proteins is involved in the development of various inflammatory and autoimmune diseases. For example, exosomes released from synovial fibroblasts of patients with rheumatoid arthritis accumulate high concentrations of membrane-bound TNF-α, exacerbating the pathology of rheumatoid arthritis [339,340]. Further, cancer-cell-derived exosomes have various immunosuppressive effects in addition to inducing apoptosis in immune cells. For example, exosomes suppress the expression of the NKG2D receptor in NK cells, which is involved in the recognition mechanism of cancer cells and reduces cancer cytotoxicity [341,342,343,344].

In contrast, when cancer-cell-derived exosomes are taken up by monocytes, the effects of TGF-β and prostaglandin E2 contained in the exosomes induce the differentiation of monocytes into bone-marrow-derived immunosuppressive cells, which release various immunosuppressive molecules such as IL-10, thereby promoting the inactivation of immunocompetent cells and the induction of regulatory T cells to suppress anti-tumour immunity [345]. Through these mechanisms, cancer cells may suppress the functions of the immune cells that attack them and promote cancer progression.

### 7.2. EV-Mediated Cytokine Enhancement and Tumour Progression

Cytokines are paracrine factors involved in the immune system where EV-mediated cytokine enhancement has been reported. For example, IFN-γ bound to its receptor on the EV surface stimulates target cells more efficiently than IFN-γ alone. Additionally, for the antagonism of CD47, a “Not Eat Me” signal is overexpressed on the surface of most tumours by a signal-regulatory protein alpha. This protein is expressed on phagocytic cells that interact with CD47-bearing EVs, inhibiting CD47 on cancer cells and thereby increasing cancer cell phagocytosis and inducing anti-tumour T cell responses [346,347,348,349]. Additionally, EVs have been shown to evade immune clearance better than artificial liposomes, likely due to the expression of CD47 on exosomal membranes. Furthermore, CD47 is overexpressed in tumours where its expression is associated with poor progression-free survival and inversely correlated with macrophage infiltration in tumour tissues loaded on exosomes, thereby promoting tumour immune evasion. The inhibition of exosome secretion or uptake via the knockdown of RAB27A, a regulator of exosome secretion, reduces the expression of CD47 in tumour cells, promotes phagocytosis by M1 macrophages in tumour tissues, and suppresses tumour progression [348]. siRNA-loaded exosomes are selectively taken up by pancreatic tumour cells, which is facilitated by enhanced micropinocytosis. This reduces oncogenic KRAS signalling and suppresses tumour growth in multiple mouse models of pancreatic cancer [349]. Therefore, increasing cancer cell phagocytosis by antagonising CD47 signalling with EVs may be an effective approach for cancer immunotherapy. Furthermore, anti-PD-L1 therapy using platelets and platelet-derived EVs has also been reported, where the PD-L1 monoclonal antibody is bound to the surface of platelets [350,351]. When these platelets are intravenously administered, they become activated and release EVs into tumour tissue. In contrast, anti-PD-L1-bound platelets are activated in the tumour microenvironment and release anti-PD-L1-bound EVs, which inhibit immune checkpoints in tumour tissue, thereby increasing the number of infiltrating CD8^+^ and CD4^+^ T cells within the tumour. In addition, the number of CD4^+^ Foxp3^+^ T cells was reduced, while the proliferation of CD8^+^ and CD4^+^ effector T cells within the tumour was enhanced. Furthermore, the administration of anti-PD-L1 antibody-conjugated platelets suppressed cancer growth and metastasis in tumour-bearing mice and significantly prolonged their survival. This suggests that platelet-secreted EVs are effective delivery carriers that inhibit immune checkpoint molecules. In addition, based on the susceptibility of EVs to macrophage uptake, anti-inflammatory therapy has been reported by loading EVs with the NF-κB binding domain (NBD), an inhibitory peptide of NF-κB, which is a transcriptional factor that promotes inflammation and delivers EVs to macrophages [352,353,354]. For this strategy, a fusion protein is prepared in which Gag, an EV inner-membrane tropic protein, is fused with NBD. The addition of Gag-NBD-loaded EVs to LPS-stimulated macrophages significantly suppressed macrophage inflammatory responses, that is, inflammatory cytokine production, nitric oxide (NO) synthase induction, and subsequent NO production [355,356]. Therefore, EVs-mediated immune responses are essential factors for tumour progression.

## 8. Conclusions

EVs derived from cancer cells contain cancer antigens, and their administration induces a cancer antigen-specific immune response, thereby exerting an anti-tumour effect. Therapeutic studies using mouse solid tumour models have shown that EV aggregates can induce anti-tumour immunity more efficiently than EVs alone and markedly delay tumour growth. When cancer-cell-derived exosomes are taken up by monocytes, the TGF-β and prostaglandin E2 contained in the exosomes induce the differentiation of monocytes into bone-marrow-derived immunosuppressive cells, which in turn release various immunosuppressive molecules, such as IL-10, thereby promoting the inactivation of immunocompetent cells and the induction of regulatory T cells to suppress anti-tumour immunity. Understanding the molecular mechanisms in which EVs-mediated immunosuppression will help to develop a novel therapeutic strategy against cancers.
